# Elixir Guarano

**Published:** 1878-02-20

**Authors:** 


### ELIXIR GUARANO.

The above article we have found to be an admir able remedy. In one case of nervous headache, in a female who had been subject to most prolonged and obstinate attacks, which being unrelieved, as a rule, by any remedy heretofore used, would continue for days, assuming, after the first day or two, a bilious character, and continuing with vomiting and suffering for a period of two and three weeks, leaving the patient utterly prostrated, by the use of Wyeth's Elixir Guarano. the second day of one of these attacks, she was relieved as by magic, since which time, near twelve months ago, she has kept the remedy in hand, and though often threatened, has in every instance aborted the attack by the use of this preparation. We must confess that the preparations of Wyeth & Co. are not only beautifully gotten up, but are efficient, pure and reliable.

Vick's Floral Guide is a very beautiful and interesting monthly journal, illustrated, and devoted to horticultural purposes, flowers, seeds, shrubbery, etc., etc. Floral Guide, quarterly, only 25 cents. Flower and Vegetable Garden, 50 cents. Address James Vick,
Rochester, N. Y.

